# Supplementary material for: Vocal and locomotor coordination develops in association with the autonomic nervous system
Source: eLife. 2019 Jul 16;8:e41853. doi: 10.7554/eLife.41853 (PMC6684270; doi:10.7554/eLife.41853)
Supplement: Transparent reporting form [file elife-41853-transrepform.docx]

***eLife’s* transparent reporting form**

We encourage authors to provide detailed information *within their submission* to facilitate the interpretation and replication of experiments. Authors can upload supporting documentation to indicate the use of appropriate reporting guidelines for health-related research (see [EQUATOR Network](http://www.equator-network.org/%20)), life science research (see the [BioSharing Information Resource](https://biosharing.org/)), or the [ARRIVE guidelines](http://www.plosbiology.org/article/info:doi/10.1371/journal.pbio.1000412) for reporting work involving animal research. Where applicable, authors should refer to any relevant reporting standards documents in this form.

If you have any questions, please consult our Journal Policies and/or contact us: [editorial@elifesciences.org](mailto:editorial@elifesciences.org).

**Sample-size estimation**

- You should state whether an appropriate sample size was computed when the study was being designed
- You should state the statistical method of sample size computation and any required assumptions
- If no explicit power analysis was used, you should describe how you decided what sample (replicate) size (number) to use

Please outline where this information can be found within the submission (e.g., sections or figure legends), or explain why this information doesn’t apply to your submission:

We followed previous studies of infant marmoset vocal behavior to determine an appropriate sample size (Liao et al. 2018; Takahashi et al. 2015; Zhang & Ghazanfar 2016;). These studies range from 6-10 animals; and so, our sample size (n=7) fits within this range.

**Replicates**

- You should report how often each experiment was performed
- You should include a definition of biological versus technical replication
- The data obtained should be provided and sufficient information should be provided to indicate the number of independent biological and/or technical replicates
- If you encountered any outliers, you should describe how these were handled
- Criteria for exclusion/inclusion of data should be clearly stated
- High-throughput sequence data should be uploaded before submission, with a private link for reviewers provided (these are available from both GEO and ArrayExpress)

Please outline where this information can be found within the submission (e.g., sections or figure legends), or explain why this information doesn’t apply to your submission:

Each infant subject (n=7) represented a biological replicate. Daily behavioral experiments represented the technical replicates, in which each subject underwent an experimental session once every 1-3 days (on average) across the first two months of development (Number of sessions: Subject 1 = 29, Subject 2 = 29, Subject 3 = 34, Subject 4 = 34, Subject 5 = 31, Subject 6 = 31, Subject 7 = 32). We included all data in the analyses.

**Statistical reporting**

- Statistical analysis methods should be described and justified
- Raw data should be presented in figures whenever informative to do so (typically when N per group is less than 10)
- For each experiment, you should identify the statistical tests used, exact values of N, definitions of center, methods of multiple test correction, and dispersion and precision measures (e.g., mean, median, SD, SEM, confidence intervals; and, for the major substantive results, a measure of effect size (e.g., Pearson's r, Cohen's d)
- Report exact p-values wherever possible alongside the summary statistics and 95% confidence intervals. These should be reported for all key questions and not only when the p-value is less than 0.05.

Please outline where this information can be found within the submission (e.g., sections or figure legends), or explain why this information doesn’t apply to your submission:

Sample sizes are described in the “Vocal Behavior Data Collection”, “Postural and Locomotor Behavior Data Collection”, and “Physiological Data Collection” sections. Statistical tests are described in the “Data Analysis” section. The outcomes of statistical tests are reported in the Results section.

(For large datasets, or papers with a very large number of statistical tests, you may upload a single table file with tests, Ns, etc., with reference to sections in the manuscript.)

**Group allocation**

- Indicate how samples were allocated into experimental groups (in the case of clinical studies, please specify allocation to treatment method); if randomization was used, please also state if restricted randomization was applied
- Indicate if masking was used during group allocation, data collection and/or data analysis

Please outline where this information can be found within the submission (e.g., sections or figure legends), or explain why this information doesn’t apply to your submission:

This study followed behavioral and biological variables across development and so subjects were naturally allocated into groups as subjects aged. Most of the data analyses used computational approaches to process the raw data (e.g., identification of call types, acoustic parameters, locomotor dynamics, and energy dynamics), and so observers were masked to age group. Identification of postural and locomotor behaviors required observers to watch video, and so it was not possible to mask age group for these specific data.

**Additional data files (“source data”)**

- We encourage you to upload relevant additional data files, such as numerical data that are represented as a graph in a figure, or as a summary table
- Where provided, these should be in the most useful format, and they can be uploaded as “Source data” files linked to a main figure or table
- Include model definition files including the full list of parameters used
- Include code used for data analysis (e.g., R, MatLab)
- Avoid stating that data files are “available upon request”

Please indicate the figures or tables for which source data files have been provided:

Source data is uploaded onto Dryad. DOI: doi:10.5061/dryad.km980bq
